# Preparation of rapid (chain-stopped) alkyds by incorporation of gum rosin and investigation of coating properties

**DOI:** 10.3906/kim-2001-56

**Published:** 2020-08-18

**Authors:** Cemil DIZMAN, Elif OZMAN

**Affiliations:** 1 İzel Kimya Research and Development Center, Kocaeli Turkey

**Keywords:** Alkyd, chain–stopped(rapid), condensation polymerization, gum rosin, paint

## Abstract

In this study, the synthesis, characterization, and properties of a short oil length chain–stopped(rapid) alkyd resin is investigated. Gum rosin modified alkyd resin (RA-GR) was prepared using soybean oil, phthalic anhydride, glycerin and gum rosin acid. An alkyd modified with benzoic acid (RA-BA) was also prepared for comparison. FTIR analyses and GPC measurements of the alkyds were used for characterization. Other properties such as the viscosity, acid value, and solid content of the final resins were determined. Separately, the synthesized resins were used in paint formulations without any changes in other parameters such as filler, airdrying agents, solvents, etc. Paints were applied to metal and glass surfaces and the effect of gum rosin was investigated by looking at touch and hard drying times, adhesion to metals and gloss changes. Compared to the benzoic acid modified resin (RA-BA), gum rosin modified resin (RA-GR) exhibited remarkable positive effects on the paint with a better adhesion to the metals, and short drying times without any loses in the glosses.

## 1. Introduction

The synthesis of new materials, which have superior properties compared to commercial equivalents, takes a step forward in trade and provides a more comfortable life for customers in all areas of chemistry. Many different polymers have been prepared by adding new functional groups or new chemicals that offer superior properties to the polymers [1,2]. Alkyds are cheap and useful commercial important polyesters produced especially for the paint and coatings market to prevent metal, wood, plastic, or other surfaces from corrosion, rusting, or other physical and chemical attacks by acting as a physical barrier. They are the most used binders with a large quantity consumed in decorative purposes. Because of the usage of cheap raw materials (bio-based oil, glycerol, and fatty acids) in their production, alkyds are inexpensive and more environmentally friendly than the other binders produced from petrol derivatives. Alkyds are the products of polyesterification reactions that occurs between alcohols modified with or without fatty acids and anhydrides [3–5].There are many kinds of alkyd derivatives with different physical and chemical features depending upon the type of oils or other constituents creating them. In general, we can categorize them into two classes according to their oil lengths or modification by different chemicals. According to their oil lengths, long (>55%), medium (45%-55%) and short oil (<45%) alkyds are produced from drying or non-drying oils. Especially long and medium oil alkyds are produced from drying oils such as, soybean, linseed, sunflower, castor, etc. In their chemical structures, there are double bonds that can be autooxidized with oxygen in air by the help of a siccative such as cobalt, zinc, calcium octoates, in order to make polymeric crosslinked networks. Short alkyds do not contain any double bonds or have a small amount of double bonds that do not create strong interactions between the polymers. Therefore, they are generally used with a second component to cure. Isocyanates are used as second components for room temperature curing of them. Melamine or nitrocellulose are useful for applications that needs higher temperatures, especially for stove applications [6–8]. As a second category, chemical modifications of alkyds are very common for the fulfillment of consumer needs in the market. For producing short oil alkyds with better touch drying times, benzoic acid or similar compounds are used. These types of alkyds are called rapid or chainstopped alkyds. Unlike other short alkyds, they can be crosslinked with a siccative like long and mediumoil length alkyds. Depending on the type of modifying agents, many different alkyds can be obtained [9]. Other researchers have applied different modifying agents to obtain novel rapid alkyds with superior properties [10–13].

Gum rosin is a bioderived chemical obtained from living pine trees.It is simply blended with or incorporated into various polymers with covalent bonds in order to increase the physical properties especially adhesion in many different areas such as the ink, adhesive, pharmaceutical, and varnish and gum industries. With an annual production of 1.2 million tons, gum rosin is one of the most abundant bioderived chemicals. The chemical structure of gum rosin having hydrocarbon-rich biomass makes it hydrophobic and hydrophenanthrene in its structure increases the thermal properties (Figure 1) [14–18].

**Figure:**
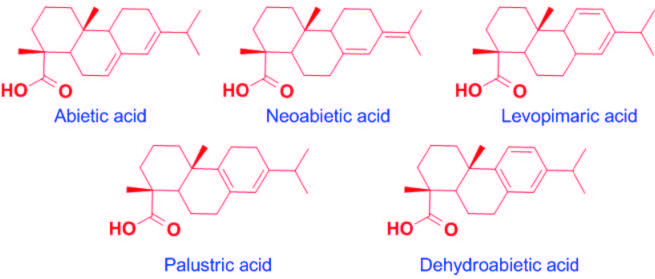
Chemical structures of major rosin acid isomers.

There are various applications in which gum rosin is blended with or employed for the synthesis of different monomeric and polymeric structures [19–24]. However, there is no detailed report on the introduction of gum rosin units into the alkyd skeleton with chemical bonds and its effect on the properties of the paint obtained therefrom. In this study, modified alkyds with or without gum rosin were prepared and used in a paint formulation separately. Comparison showed that the paint prepared from the alkyds with gum rosin has superior physical properties (adhesion, gloss and touch, and harddrying time) as compared to the paints from benzoic acid modified alkyd.

## 2. Experimental

### 2.1. Materials

Soybean oil (SO) (Samyağ Oil Corp., İstanbul, Turkey), glycerin (99%, Celmark International Inc., Orlando, FL, USA), lithium hydroxide (LiOH) (Sorel Chemicals, İstanbul, Turkey), phthalic anhydride (99%, Petkim Petrokimya Holding Corporation, İzmir, Turkey), benzoic acid (Kolsuzlar Chemistry Industry and Trade Inc., İstanbul, Turkey),and gum rosin (Resin Chemistry Trade Limited Company, İstanbul, Turkey) were used without any further purifications. Cobalt octoate, zinc octoate, and calcium octoate were bought from EGE dry company and used with any further purification.BYK 104S was bought from BYK. All other materials were used without any further purification.

### 2.2. Synthesis

#### 2.2.1. Synthesis of chainstopped (rapid) alkyd with gum rosin acid (RA-GR)

In the first step, the production of monoglycerides from soybean oils were achieved by transesterification reaction. To a 1000 mL, 5 necked roundbottomed flasks fitted with a condenser, nitrogen inlet, an overhead mechanical stirrer, and a Dean and Stark trap were added soybean oil, glycerin, gum rosin acid, lithium hydroxide (LiOH) and a little bit of xylene as an azeotropic solvent to make the water out easily. The heat was gradually increased to the 240 °C and applied for about 3 h at this temperature to achieve full alcoholysis. Alcoholysis reaction was monitored by methanol test. Methanol does not dissolve the oil but monoglycerides are soluble in it. If 1 part resin in 3 parts methanol is totally dissolved, the transesterification reaction is complete. In the second step, the reaction temperature was decreased to 160 °C and phthalic anhydride was added in one pot. Then, the reaction temperature was arranged to 220 °C and monitored at this temperature by controlling the acid and viscosity at regular intervals. When acid value was decreased below 15, the reaction was ended.

**FT-IR (ATR, cm^-1^):** 3463(-OH), 3070 (Ar), 2923(-CH_3_), 2854(-CH_2_), 1257 and 1122 (C-O-C) and 1064 (Ar).

#### 2.2.2. Synthesis of chain-stopped(rapid) alkyd with benzoic acid (RA-BA)

The same procedure as in the synthesis of the RA-GR was applied to produce benzoic acid modified alkyd resin (RA-BA). Only difference was the addition of benzoic acid instead of gum rosin acid in the second step of the reaction.

**FT-IR (ATR, cm^-1^):** 3498(-OH), 3070 (Ar), 2923(-CH_3_), 2854(-CH_2_), 1253 and 1114 (C-O-C) and 1064 (Ar).

### 2.3. Equipment

FTIR spectra were measured with JASCO FT/IR-4200 with ATR (JASCO Corp., Tokyo, Japan). Spectra were obtained at mid-IR region (ca. 4000–700 cm^-1^) at a resolution of 4 cm^-1^ with 16 scans (Spectra Manager II software, JASCO Corp.). Molecular weights and polydispersity indexes of the polymers were measured by gel permeation chromatography (GPC) employing an Agilent 1100 instrument equipped with a differential refractometer by using tetrahydrofuran (THF) as the eluent at a ?ow rate of 1 ml min^-1^ at 30 °C. Molecular weights were determined by using polystyrene standards. Brookfield viscosity was measured by Brookfield viscometer (RVDV-I Prime, 25 °C, spindle SC4-21, 50 rpm). Crosscut adhesion test kit CC2000 from TQC Sheen B.V. (Capelleaan den IJssel, Netherlands) was used to test the adhesion of dry coatings on their substrate. The brightness of the films was determined using a Novo-Gloss Trio glossmeter.

### 2.4. Preparation and characterization of paint formulations

A simple paint recipe was applied for both alkyds separately. White paints were achieved in both cases by using the same amount of resins, pigments and other ingredients in the formula. For mixing all ingredients in the paint, a high–speed mixer was used, and fineness of grinding was followed by using a grindometer. Paints were applied to the glass and metal surfaces by using a film applicator with 90 micrometer thickness. Adhesion, touch, drying time, and gloss properties of the paints were determined by using metal surfaces. Glass panels were used for determination of hard drying times by drying time recorder.

### 2.5. Preliminary tests for the prepared resin

Drying agents: Cobalt, calcium and zinc octoates were used as driers. After grinding the pigments, the siccatives were added and mixed by the help of high–speed mixer.

Drying times: Touch drying times were checked in regular intervals by applying a force with fingertip to a painted surface on a metal panel. Hard drying time was determined by drying time recorder. A paint wasapplied to a glass panel and the glass panel was putted on the machine. The scratching ending on glass panels shows the complete dry time.

Adhesion: After keeping the paint applied on the surface of a metal panel for about one day crosscut adhesion method was applied. In the adhesion test the panel was sliced with a 1 mm gap between 4 vertical and 4 horizontal lines. Cellophane tape was applied to whether or not the paint was removed from the surface to get the adhesion degree.

## 3. Results

In practical applications, the paint prepared from the chainstopped (rapid) alkyds is applied on a surface at room temperature. The autooxidation process with the help of oxygens in the air is very fast and normally 5–120 minis enough for surface (touch) drying for chainstopped alkyds. But the problem in this type of paint is the long time needed for hard drying. One day is not sufficient for complete dry. Therefore, it is important to shorten the time needed for harddrying completion and touchdrying. But the problem in the paints is that if the time for drying is shortened, the paint gloss degree gets worse. When benzoic acid is used for modification it is not possible to make the harddrying time shorter, whereas the touchdrying time is sufficiently short. In almost all cases benzoic acid provides hardness due to the aromatic rings but there existed ester functionalities sourced from the reaction of alcohols and benzoic acid groups that made the alkyd resin flexible. Also, benzoic acid reacts with hydroxyl groups, which leads to a decrease in the number of reactive hydroxyl functional groups.Hence, benzoic acid addition prevents gelation. By increasing the amount of benzoic acid low molecular weight alkyds may be obtained. Benzoic acid modified alkyds suffer from long hard drying times whereas they have short touch drying times. Additionally, the final (or complete) hardness does not fulfill the customer needs in many situations. To overcome this problem, some alcohols with high glass transition temperatures such as pentaerythritol or trimethylolethane (TME) were used instead of glycerin. But these materials are too expensive, and that increases the price of the final alkyds. Another suggestion is to add different chemicals instead of benzoic acid. In our case, gum rosin was used instead of benzoic acid to make alkyds harder and to decrease the touch and harddrying times. Without changing the amounts of soybean oil, glycerin, and phthalic anhydride, benzoic, and gum rosin modified chainstopped alkyds were synthesized separately by condensation reaction. The overall procedure was shown in Scheme. The characterization of the alkyds was carried out by using FTIR ATR and the effect of benzoic and gum rosin acids on the molecular weight of the polymers was followed by gel permeation chromatography (GPC). Finally, alkyds with benzoic acid (RA-BA) and gum rosin (RA-GR) were used in a paint recipe separately in order to determine the effect of the chemical replacement on the final coatings. The paints contain some driers that help autooxidation with air to obtain network structures after spontaneous evaporation of the solvents at room temperature.

**Scheme:**
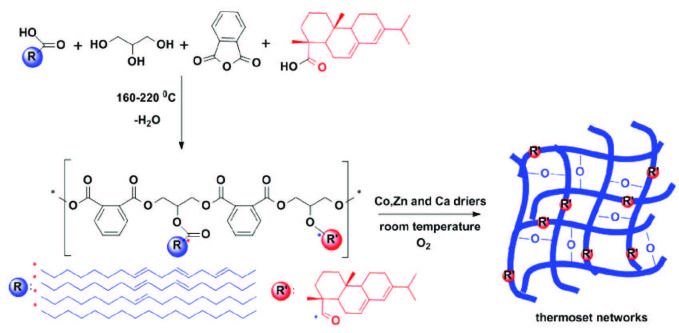
Synthetic route for the gum rosin modified rapid (chain-stopped) alkyd (RA-GR) and coating obtained therefrom.

Ingredient amounts used for the synthesis of RA-BA and RA-GR alkyds are given in Table 1. While the addition of the benzoic and gum rosin acids in the reactions the molar amount of the phthalic anhydride was decreased as the same proportion as the added (benzoic or gum rosin) acids. To keep the stoichiometry of reaction (OH/COOH ratio) the same, molar amount of the gum rosin acid was kept the same as the molar amount of the benzoic acid. By this way, the effect of benzoic and gum rosin acids on the molecular weight and viscosity of the alkyds were investigated without any change in reaction stoichiometry. After reaction of benzoic or gum resin with some hydroxyl groups, the number of hydroxyl groups that can easily react with phthalic anhydride or acid groups decreases. Bulky benzoic or gum rosin moieties may be responsible for that the bulky groups inhibit the functional groups react with each other easily and decreases the possibilities of the hyperbranching that is responsible for the high viscosities and molecular weights. Since the chemical structure of gum rosin structure is bigger than benzoic acid, gum rosin modified alkyd (RA-GR) has more hyperbranching that leads higher viscosities compared to benzoic acid modified alkyds (RA-BA).

**Table 1:** Ingredients of the modified alkyds.

Ingredients (g)	Soybean oil	Glycerin	LiOH	Phthalic anhydride	Benzoic acid	Gum rosin acid
RA-BA	120	156	0.1	250	50	-
RA-GR	120	156	0.1	250	-	123.8

The molecular weight characteristics of the benzoic and gum rosin acid modified alkyds were also monitored by GPC analysis (Table 2). The molecular weight of the RA-GR alkyd was higher than the RA-BA. This may be due to the higher molecular weight of the gum rosin acid as compared to benzoic acid.

**Table 2:** Molecular weight characteristics of the modified alkyds.

Sample	M^a^_w_(g/mol)	M_w_/M_n_a
RA-BA	4.169	2.99
RA-GR	8.165	4.48

^a^Number average of molecular weights and molecular weight distributions were determined by GPC equipment based on polystyrene standards.

**Figure 2:**
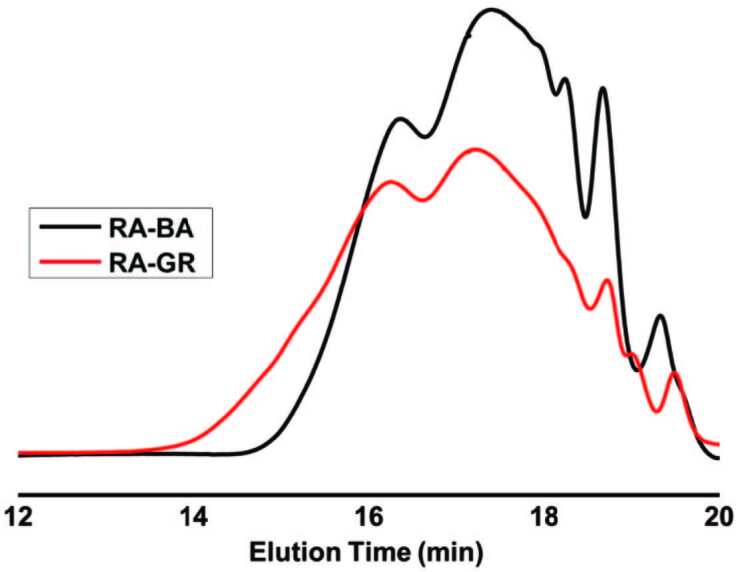
GPC traces ofbenzoic acid modified alkyd (RA-BA), and gum rosin acid modified alkyd (RA-GR).

No oligomers or degradation products with small molecular weights were detected during the whole process showing that all monomers were fully reacted as shown in Figure 2. The viscosity of the RA-GR (30.000 mPa.s) is much higher than the RA-BA (600 mPa.s). This may be due to the higher molecular weight of the RA-GR as compared to the RA-BA, resulting in the higher viscosity and larger polydispersity as shown in Table 2.

**Figure 3:**
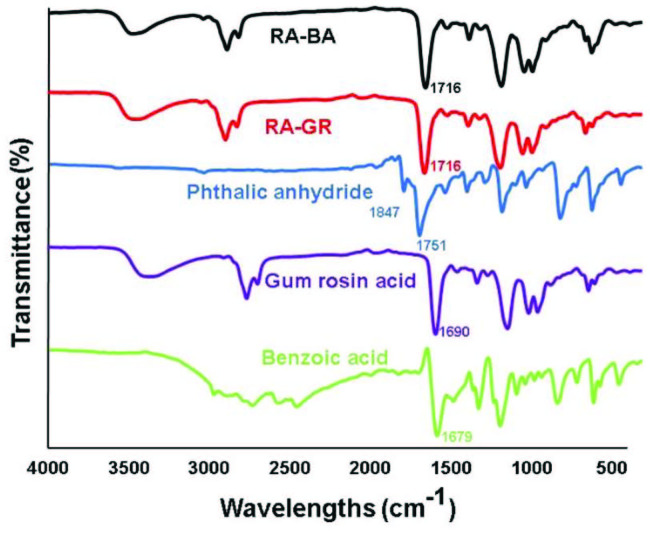
FT-IR ATR spectra of the benzoic acid modified alkyd (RA-BA), and gum rosin acid modified alkyd (RA-GR).

The synthesis was also monitored by FT-IR ATR analysis following characteristic ester peaks arising from the reaction of alcohols and acids or anhydrides at 1716 cm^-1^. The peak related to anhydride at 1847 and 1751 cm^-1^ was totally disappeared, showing that all phthalic anhydride reacted with alcohols. In the case of gum rosin modification, the acid peak at 1690 cm^-1^ of the gum rosin was shifted to 1716 cm^-1^ as in the case of benzoic modification in which the acid peak at 1679 cm^-1^ was shifted to 1716 cm^-1^, showing the successful esterification (Figure 3.).

Physicochemical characteristics of the RA-BA and RA-GR alkyds were given in Table 3. The fact that the iodine value of RA-GR is almost twice that of RA-BA explains why the drying time of the paint prepared from gum rosin acid has a shorter drying time than the paint prepared from benzoic acid modified alkyd.

**Table 3:** General properties of the RA-BA and RA-GR alkyds.

Characteristics	RA-BA	RA-GR
Acid value (100% solid), mgKOH/g	10.4	12.4
Iodine value, gI_2_/100 g	21	41.5
Viscosity, Brookfield at 25 °C, mPa.s	600	30,000
Nonvolatile, %	60	60

Paint properties of the RA-BA and RA-GR alkyds were determined by applying the paint therefrom to the metal and glass surfaces. A typical gloss paint formulation was shown in Table 4 and similar paint formulations were applied for each alkyd.

**Table 4:** A typical gloss paint recipe.

Ingredients	Amount (g)
Resin (60% solid in Toluene)	60
Titanium dioxide (TiO_2_)	22.3
Dispersant (BYK 104S)	2.7
Solvent (Toluene)	13
Antiskinning agent (Methyl ethyl ketoxime)	1
Co (6%)	0.3
Zn (24%)	0.74
Ca (4%)	1.34

The paints were applied to glass and metal surfaces. The properties of the coated paints were shown in Table 5. Paint with gum rosin modified alkyd showed better drying times. This may be due to the fact that gum rosin has double bonds that make alkyds more cross-linked with each other. While autooxidation process takes place with oxygen in air, the radicals are formed in double bonds in the oily part. These radicals may attack to double bonds on the gum rosin structure that leads to increase the number of crosslinked bonds. This increase in the crosslink density makes the films stronger and increases the physical properties of the final coatings. Also, hydrophobic nature of the gum rosin chemical structure may help the drying times shorter due to the fact that the moisture increases the drying times of the paints. Although the drying times of the RA-GR based paint were short, the gloss values were very high. This may be due to the chemical nature of gum rosin. Some of the double bonds in the chemical structure of gum rosin disappear when auto-oxidation process occurs as mentioned above. This makes the gum rosin structure with no double bonds similar to cycloaliphatic chemical structure that is very useful in making transparent and bright coating films [25,26].

**Table 5:** Properties of the paints.

Parameters	RA-BA	RA-GR
Surface dry (min)	90	15
Dry through (h)	19.5	20
Gloss values (60 °)	86	85

### 3.1. Discussion

In conclusion, alkyds from gum rosin and benzoic acids were successfully prepared by condensation reaction and paint properties of obtained therefrom were investigated. By auto-oxidation of double bonds with the help of some siccatives, thermoset crosslinked networks were obtained on glass and metal surfaces. The gum rosin moieties contribute to enhance the drying times and gloss values. Also, the adhesiveness to metals was increased by addition of gum rosin by comparing to benzoic acid units. Gum rosin is a good candidate to be used in alkyd formulations enhancing the paint properties (drying times without losing any glossiness) prepared therefrom.
